# Outcomes of Neurorehabilitation Among Patients With Prolonged Disorders of Consciousness

**DOI:** 10.7759/cureus.38816

**Published:** 2023-05-10

**Authors:** Mohammed Saif, Shatha A Sharbatti, Anas Nemmar, Sharanya S Kumar, Krishna Prasad, Asma M Khan, Iman Khadar, Sharmila Banu

**Affiliations:** 1 Medicine, Gulf Medical University, Ajman, ARE; 2 General Practice, Thumbay University Hospital, Ajman, ARE; 3 Physical Medicine and Rehabilitation, Thumbay University Hospital, Ajman, ARE

**Keywords:** pdoc, deformity, muscle girth, comatose, superficial sensation, neurorehabilitation, prolonged disorders of consciousness

## Abstract

Background: The impact of neurorehabilitation on patients with prolonged disorders of consciousness (PDOC) is not well known. We assessed the range of motion (ROM), muscle girth and power, level of consciousness, development of musculoskeletal deformity, and superficial sensation.

Methods: A retrospective observational record-based study was done, which included the data of patients diagnosed with PDOC admitted at Thumbay Physical Therapy & Rehabilitation Hospital, Ajman, UAE, between 2020 and 2022. Data on the “range of motion”, “muscle girth and power”, “level of consciousness”, “development of musculoskeletal deformity”, and “superficial sensation” were collected and analyzed. The SPSS software version 27 (IBM Corp., Armonk, NY, USA) was used for analysis. The chi-square test was used to assess association, and the t-test was used to test the mean difference.

Results: We assessed the data of 21 patients with PDOC. The superficial sensation was found to have increased significantly (p<0.025). There was a decrease in the proportion of patients with musculoskeletal deformities during the follow-up period. The ROM, muscle girth, and muscle power were also preserved without significant deterioration. However, the level of consciousness measured by the Glasgow coma scale (GCS) showed no improvement.

Conclusions: Our research showed that neurorehabilitation significantly improves superficial sensation and prevents the development of musculoskeletal deformities. However, the mean level of consciousness remained the same. There was also no decrease in ROM. Both muscle girth and power were preserved over two years.

## Introduction

The brain automatically starts regaining lost capability as it seeks to restore itself. Even after injury, the human brain is incredibly malleable and constantly strives to adapt to new situations. The primary goals of neurorehabilitation are to achieve the highest level of functional autonomy, including physical, cognitive, and behavioral independence. After experiencing a neurological impairment, neurorehabilitation seeks to return a person to the greatest extent of their abilities. Diagnosis in medical rehabilitation includes evaluating medical problems and how they affect functionality. It often takes a team effort and combines diagnostic evaluations from other disciplines to produce a range of rehabilitative therapy plans and objectives. It aids in identifying deficiencies and establishing treatment objectives by advancing our understanding of neurological impairments and syndromes as they relate to functional incapacity [1]. 

Research by Gurin et al. on the outcomes of early neurorehabilitation among brain injury patients with COVID-19 found that more than half of the study patients achieved positive outcomes, including reaching a minimally conscious state (MCS) or better, before being discharged [2]. A similar study by Edlow et al. assessed the outcome of rehabilitation therapies such as pharmacologic, electromagnetic, sensory, mechanical, and regenerative and found a gap in knowledge for effective therapy for unconscious patients [3]. The study by Thibaut et al. on the impact of physical therapy on spasticity and muscle contraction among patients with disorders of consciousness (DOC) found that such therapies significantly reduced spasticity and muscle contracture [4]. A study from Saudi Arabia showed that the functional outcome of patients suffering from traumatic brain injury (TBI) is affected by the severity of the disability at admission and the time between injury and admission [5].

Data is limited related to the outcome of neurorehabilitation in patients with prolonged disorders of consciousness (PDOC) in the UAE. This study will shed light on the effects of neurorehabilitation on the prognosis of patients with PDOC for more than two years.

Studies have shown the positive impact of neurorehabilitation in patients with acute DOC (less than four weeks) [2,4]. No study exists in the UAE regarding the outcomes of neurorehabilitation amongst patients with DOC for more than four weeks. This study will help medical professionals have an in-depth understanding of the outcomes of neurorehabilitation in the recovery or management of patients with PDOC.

This article was previously posted to the Research Square preprint server on February 3, 2023.

## Materials and methods

Research design and study population

This is a retrospective observational record-based study. Patients clinically diagnosed as being in a vegetative state for more than four weeks were identified as having PDOC. We studied them for two years as they were admitted to Thumbay Physical Therapy & Rehabilitation Hospital for long-term care between 2020 and 2022. 

Each of the patients included in the study was monitored regularly. They all had scheduled daily physiotherapy sessions to prevent musculoskeletal deformities and increase limb mobility. The daily physiotherapy sessions included passive mobility and sensory stimulatory therapies such as positional exercises, passive range of motion (ROM), passive stretching, active assisted, and chest physiotherapy. Neurologists supervised each patient to monitor and assess their health regularly. Pain and temperature stimuli were used to assess the superficial sensation of the patients by observing their reactions, such as flickering of the eye and withdrawal of limbs during the manoeuvers. The manual muscle testing scale was used to assess muscle power. Although the Glasgow coma scale (GCS) may not be an accurate measure of the level of consciousness in the rehabilitation state, it was the only parameter of assessment available in the patient's medical record to help understand their level of consciousness. Every patient had a tailored diet prepared by the nutritionist to ensure healthy progress. All patients were assigned a nurse to monitor and provide daily care. All methods were performed under the relevant guidelines and regulations of the Declaration of Helsinki. 

Inclusion and exclusion criteria

The inclusion criteria for this study involved male and female participants from all age groups with a confirmed diagnosis of PDOC. However, patients with incomplete medical records were excluded from the study.

## Results

The data of 21 patients with PDOC were analyzed. Table 1 shows the difference between the proportion of musculoskeletal deformity before and after receiving rehabilitation care. Neurorehabilitation helped prevent the development of musculoskeletal deformities such as bone deformity leading to flexion and extension abnormality. It also decreased the proportion of patients with musculoskeletal deformities during the follow-up period of two years. 

**Table 1:** Presence of musculoskeletal deformity at the beginning and end of the study period N: Number of participants

Presence of musculoskeletal deformity	Present		Absent		P-value
	N	%	N	%	
Start of study period	15	71.4	6	28.6	0.267
End of study period	12	57.1	9	42.9	

Table 2 shows the difference between the proportion of superficial sensations before and after receiving rehabilitation care. We assessed the superficial sensation with pain and temperature stimuli and identified patients' reactions with flickering of eyes and withdrawal of limbs in response to the maneuvers. When assessed for superficial sensation two years before, we found that more than 20% of patients had impaired sensation. There was a significant increase in superficial sensation over two years of neurorehabilitation.

**Table 2:** Presence of superficial sensation at the beginning and end of the study period N: Number of participants

Presence of superficial sensation	Present		Absent		P-value
	N	%	N	%	
Start of study period	16	76.2	5	23.8	0.025
End of study period	21	100	0	0	

Table 3 shows the mean difference between the arm, forearm, thigh, and calf muscle girth before and after the study period. Neurorehabilitation helped prevent the deterioration of muscle. Muscle wasting is a significant finding among most paralyzed patients. However, the sample patients did not have any significant muscle wasting.

**Table 3:** Muscle girth measurement at the beginning and end of the study period SD: Standard deviation

Muscle girth			Mean (cm)	SD (cm)	P-value	95% confidence interval	
						Lower	Upper
Arm	Right	Start of study period	24.239	5.424	0.617	-1.770	1.081
		End of study period	24.583	5.796			
	Left	Start of study period	24.722	5.557	0.794	-1.748	1.359
		End of study period	24.916	5.076			
Forearm	Right	Start of study period	19.938	5.141	0.014	-2.425	-0.307
		End of study period	21.305	5.366			
	Left	Start of study period	19.194	5.233	0.139	-1.967	0.300
		End of study period	20.027	5.117			
Thigh	Right	Start of study period	30.429	6.094	0.186	-4.578	0.966
		End of study period	32.235	8.357			
	Left	Start of study period	31.088	6.280	0.062	-5.075	0.134
		End of study period	33.559	7.206			
Calf	Right	Start of study period	21.933	5.437	0.125	-2.719	0.363
		End of study period	23.111	4.720			
	Left	Start of study period	21.933	5.743	0.046	-3.275	-0.024
		End of study period	23.583	4.768			

Table 4 shows the mean difference between the level of consciousness before and after receiving rehabilitation care. Patients' level of consciousness was maintained over the two years of the study. The mean GCS score at the start and end of the study duration remained similar, with no evidence of any significant deterioration.

**Table 4:** Level of consciousness at the beginning and end of the study period GCS: Glasgow coma scale, SD: Standard deviation

Level of consciousness (GCS)	Mean	SD	P-value	95% confidence interval	
				Lower	Upper
Start of study period	6.90	1.513	0.237	-0.607	-0.154
End of study period	6.90	1.338			

Table 5 shows the mean difference between the ROM before and after receiving rehabilitation care. Neurorehabilitation helped prevent significant deterioration of muscle power. The muscle was preserved for most patients over two years of the study.

**Table 5:** Range of motion measurement at the beginning and end of the study period ROM: Range of motion, SD: Standard deviation, PRS: Professional rehabilitation services

Range of motion				Mean (°)	SD (°)	P-value	95% confidence interval	
Elbow	Flexion	Right	Start of study period	65.71	42.346	0.923	-21.343	19.438
			After PRS	66.67	52.090			
		Left	Start of study period	78.00	43.724	0.483	-13.056	26.675
			End of study period	71.19	51.012			
	Extension	Right	Start of study period	60.38	51.948	0.187	-17.675	3.675
			End of study period	67.38	53.095			
		Left	Start of study period	60.38	52.664	0.733	-25.796	18.462
			End of study period	65.05	55.354			
Knee	Flexion	Right	Start of study period	49.05	49.940	0.167	-6.064	32.730
			End of study period	35.71	43.540			
		Left	Start of study period	46.81	46.149	0.480	-11.580	23.770
			End of study period	40.71	42.728			
	Extension	Right	Start of study period	29.00	44.827	0.441	-22.882	10.382
			End of study period	35.00	43.818			
		Left	Start of study period	24.90	36.407	0.063	-29.569	0.869
			End of study period	38.33	43.684			

Table 6 shows the mean difference between the muscle power scored by the MMT before and after receiving rehabilitation care. Neurorehabilitation helped prevent significant loss of muscle power and strength. The muscle power and strength were maintained over the two years, which helped prevent comorbidities. The MMT scale was used to assess the muscle power; however, since the patients had minimal GCS scores, the MMT score was an average of 1 which signifies no resultant motion but with trace contractile function.

**Table 6:** Muscle power assessment at the beginning and end of the study period MMT: Manual muscle test, SD: Standard deviation

Muscle power				Mean (MMT score)	SD (MMT score)	P-value	95% confidence interval	
							Lower	Upper
Shoulder	Flexion	Right	Start of study period	1.43	1.630	0.149	-0.205	1.252
			End of study period	0.90	0.944			
		Left	Start of study period	1.19	0.981	1.000	-0.432	0.432
			End of study period	1.19	0.981			
	Extension	Right	Start of study period	0.95	1.532	0.196	-0.240	1.097
			End of study period	0.52	0.602			
		Left	Start of study period	1.14	1.558	0.196	-0.240	1.097
			End of study period	0.71	0.784			
Hip	Flexion	Right	Start of study period	1.29	1.765	0.242	-0.382	1.430
			End of study period	0.76	0.831			
		Left	Start of study period	1.14	1.558	0.618	-0.593	0.974
			End of study period	0.95	0.805			
	Extension	Right	Start of study period	0.86	1.526	0.319	-0.347	1.014
			End of study period	0.52	0.602			
		Left	Start of study period	0.90	1.513	0.176	-0.208	1.065
			End of study period	0.48	0.512			
Knee	Flexion	Right	Start of study period	1.24	1.729	0.341	-0.488	1.345
			End of study period	0.81	0.928			
		Left	Start of study period	1.00	1.517	1.000	-0.852	0.852
			End of study period	1.00	1.000			
	Extension	Right	Start of study period	0.85	1.565	0.804	-0.730	0.930
			End of study period	0.76	0.944			
		Left	Start of study period	1.30	1.750	0.550	-0.731	1.331
			End of study period	0.95	1.024\			

## Discussion

Neurorehabilitation is an essential aspect of patient care, particularly for those who have suffered TBIs and have DOC. Hence, understanding the impact of neurorehabilitation on patients' prognoses can help healthcare professionals provide better care and improve outcomes [2,4]. As such, we conducted a research study to evaluate the impact of neurorehabilitation on patients with DOC, focusing on the assessment of superficial sensation, muscle girth and power, and the GCS score. This study is the first to be conducted in the United Arab Emirates, and we believe it will contribute significantly to understanding neurorehabilitation and improve patient outcomes.

Association between neurorehabilitation and superficial sensation

Our study showed that there was a significant improvement in superficial sensation. Patients were regularly given sensory stimulation to assess for any improvement or deterioration. At the end of the study period, we noted that all patients could respond when a superficial sensory stimulus was given, which was assessed with pain and temperature stimuli identifying patients’ reactions during the maneuvers such as flickering of the eye or withdrawal of limbs.

Research by Heidler shows that multisensory interventions improved sensory deficits in patients with brain injury in an acute scenario. However, it could not conclude similar results for patients with DOC on a long-term basis [6].

Similar findings were present in research done by Dolce et al., where they found improvement in the sensory abilities of the patients measured by visual pursuit response. Patients with traumatic injury had better outcomes than non-traumatic vascular injury subjects. However, this study was conducted among patients with acute onset of brain injury [7]. Limited and sparse data exist on the effects of sensory stimulation on patients with DOC for a prolonged duration.

Association between neurorehabilitation and changes in the level of consciousness in an inpatient setting

Survivors of a TBI can experience disorders in their level of consciousness, ranging from a vegetative state to MCS. Inpatients who started rehabilitation without command-following earlier regained their independence in mobility, self-care, awareness, and cognition 10 years post-injury [8].

Our study assessed 21 patients paralyzed with impaired consciousness for over two years with an average GCS score of 7. These patients were not fully capable of independent mobility, and their GCS score was the same or slightly improved. This predisposed them to a higher risk of developing musculoskeletal deformity.

A retrospective cohort study done in a neurorehabilitation center in southern Germany showed that of those who suffered from TBI with DOC, 37.2% of participants recovered from MCS, and at least 16.5% gained functional recovery by week seven post-injury [9].

A prospective observational study conducted in a neurological rehabilitation center in Germany screened for the level of consciousness in patients with brain injury after undergoing neurorehabilitation during hospitalization and one-year post-discharge. The results showed that patients with MCS upon admission showed better outcomes after discharge. They were more likely to recover within one-year post-injury in terms of their level of consciousness [10]. 

Association between neurorehabilitation and changes in muscle power and muscle mass

Decreased mobility during hospitalization, both for an acute period (less than three weeks) and for a long-term period (more than three weeks), leads to a significant increase in morbidity, disability, and, if severe, even mortality from hospital-acquired infections especially in older bed-ridden patients [11].

Neurorehabilitative measures are paramount in preserving muscle girth and joint mobility. Our research study assessed the muscle power and girth of arms, forearms, thighs, and calves post-neurorehabilitation. We found both muscle power and girth to have been maintained and even slightly increased among our participants in the two years of the study. 

A study by de Asteasu et al. in an acute geriatrics unit in Spain shows significant enhancements in the older population's muscle power and balance in their upper/lower limbs and functional capacity after an individualized physical exercise program [12]. An eight-week randomized-controlled study done in Slovenia assessed 25 male patients with TBI. It showed significant muscle strength, endurance, attentiveness, and cardiovascular system improvement after undergoing endurance exercises such as aerobic exercise [13]. A four-week prospective study at stroke rehabilitation units in Japan conducted by Irisawa et al. assessed muscle strength, mass, and its effect on activities of daily life (ADL). It showed improved muscle strength, activation, and ADL post-stroke rehabilitation [14].

While these studies support our research findings, it's essential to consider some limitations that may have impacted our analysis. These limitations include our small sample size and varying age groups. Additionally, we took into account patients' nutritional status and any changes in their dietary plan, as these factors can also impact muscle mass, quality, and strength.

Outcomes of neurorehabilitation on ROM, passive mobility, and development of musculoskeletal deformity

Contractures are most commonly observed after an acquired brain injury. They are one of the main reasons to limit functional capability, mobility, balance, and sensory impairments and cause pain. These can also cause a delay in motor recovery, poor rehabilitation results, and prolonged hospitalization. Passive stretching and motor training have been widely used to prevent this complication [15]. Our study revealed that a patient's ROM in the body's major joints was preserved without any statistically significant deterioration. The neurorehabilitative procedures helped prevent the development of spasticity and rigidity. 

A study conducted in the Hospital of the Lithuanian University of Health Sciences evaluated the impact of physical rehabilitation on motor recovery and changes in mental status in those who sustained TBIs. It showed that recovery of motor function was more significant in those who suffered from a coma for up to one week compared to those who were in a coma longer. It was also seen that advanced hand movements along with walking ability are the most impaired post-rehabilitation, but with a prolonged duration of rehabilitation, these can be overcome [16].

Another study conducted by Hosseini et al. in Iran analyzed the impact of early passive exercises on motor function in stroke patients within three months of the event. A dramatic improvement was observed within the first month of physical rehabilitation in the upper extremities. In contrast, the lower extremities improved in the third month. The control group achieved the same improvement in both extremities by the third month [17].

Ankle muscles have been considered the most significant contributor to functional gait, balance, and coordination purposes in chronic stroke patients. A study was conducted at South Korea National Rehabilitation Center to understand the effects of biaxial ankle muscle training (AMT). It includes passive stretching and contractions and active-resistive strengthening exercises via visual feedback on a chronic stroke patient's walking ability, balance, and ankle muscle efficiency. It shows increased ankle muscle strength and efficiency, especially during gait. It demonstrates that targeted strengthening training exercises are more crucial for the distal parts of the lower limbs than the whole lower limb training [18].

Patients who have been paralyzed for a long time may have a higher risk of developing musculoskeletal deformities. However, with regular neurorehabilitative therapy, there was no increase in the development of musculoskeletal deformities. Despite preserving and maintaining joint mobility and muscle tone, the mean GCS of our participants remained the same.

## Conclusions

Our research showed that neurorehabilitation significantly improves superficial sensation and prevents the development of musculoskeletal deformities in patients with DOC for a prolonged duration (>4 weeks). However, the mean level of consciousness remained the same. There was also no decrease in ROM. The muscle girth and power were preserved throughout the study for two years. 

Neurorehabilitation aims to retrain the nervous system, helping the patient regain muscle and sensation control. Through exercises, therapies, and other interventions, neurorehabilitation can promote the growth and strengthening of muscles, reduce the risk of muscle atrophy and deformity, and improve overall function. The importance of neurorehabilitation cannot be overstated, as it can significantly improve the quality of life for patients with neurological conditions and injuries and help them regain their independence. As our population ages and the incidence of neurological conditions increase, the need for effective neurorehabilitation will only continue to grow, making it an essential field of therapy for the future.

## References

[1] Katz DI, Dwyer B (2021). Clinical neurorehabilitation: using principles of neurological diagnosis, prognosis, and neuroplasticity in assessment and treatment planning. Semin Neurol.

[2] Gurin L, Evangelist M, Laverty P (2022). Early neurorehabilitation and recovery from disorders of consciousness after severe COVID-19. Neurocrit Care.

[3] Edlow BL, Sanz LR, Polizzotto L (2021). Therapies to restore consciousness in patients with severe brain injuries: a gap analysis and future directions. Neurocrit Care.

[4] Thibaut A, Wannez S, Deltombe T, Martens G, Laureys S, Chatelle C (2018). Physical therapy in patients with disorders of consciousness: impact on spasticity and muscle contracture. NeuroRehabilitation.

[5] Ullah S, Bin Ayaz S, Moukais IS, Qureshi AZ, Alumri T, Wani TA, Aldajani AA (2020). Factors affecting functional outcomes of traumatic brain injury rehabilitation at a rehabilitation facility in Saudi Arabia. Neurosciences (Riyadh).

[6] Heidler MD (2008). Effects of multisensory stimulation interventions in brain-damaged patients. Rehabilitation (Stuttg).

[7] Dolce G, Arcuri F, Carozzo S (2015). Care and neurorehabilitation in the disorder of consciousness: a model in progress. Sci World J.

[8] Malone C, Erler KS, Giacino JT (2019). Participation following inpatient rehabilitation for traumatic disorders of consciousness: a TBI model systems study. Front Neurol.

[9] Klein AM, Howell K, Vogler J, Grill E, Straube A, Bender A (2013). Rehabilitation outcome of unconscious traumatic brain injury patients. J Neurotrauma.

[10] Boltzmann M, Schmidt SB, Gutenbrunner C, Krauss JK, Höglinger GU, Rollnik JD (2022). One-year outcome of brain injured patients undergoing early neurological rehabilitation: a prospective observational study. BMC Neurol.

[11] Rosman M, Rachminov O, Segal O, Segal G (2015). Prolonged patients' in-hospital waiting period after discharge eligibility is associated with increased risk of infection, morbidity and mortality: a retrospective cohort analysis. BMC Health Serv Res.

[12] de Asteasu MLS, Martínez-Velilla N, Zambom-Ferraresi F (2020). Changes in muscle power after usual care or early structured exercise intervention in acutely hospitalized older adults. J Cachexia Sarcopenia Muscle.

[13] Romanov R, Mesarič L, Perić D, Vešligaj Damiš J, Petrova Filišič Y (2021). The effects of adapted physical exercise during rehabilitation in patients with traumatic brain injury. Turk J Phys Med Rehabil.

[14] Irisawa H, Mizushima T (2022). Assessment of changes in muscle mass, strength, and quality and activities of daily living in elderly stroke patients. Int J Rehabil Res.

[15] Leung J, Harvey LA, Moseley AM (2013). An intensive programme of passive stretch and motor training to manage severe knee contractures after traumatic brain injury: a case report. Physiother Can.

[16] Lendraitienė E, Petruševičienė D, Savickas R, Žemaitienė I, Mingaila S (2016). The impact of physical therapy in patients with severe traumatic brain injury during acute and post-acute rehabilitation according to coma duration. J Phys Ther Sci.

[17] Hosseini ZS, Peyrovi H, Gohari M (2019). The effect of early passive range of motion exercise on motor function of people with stroke: a randomized controlled trial. J Caring Sci.

[18] Cho J, Lee W, Shin J, Kim H (2021). Effects of biaxial ankle strengthening on muscle efficiency during gait in chronic stroke patients: a randomized controlled pilot study. Gait Posture.

